# Long‐term survival and association of neoadjuvant chemotherapy with or without immunotherapy in resectable esophageal squamous cell carcinoma

**DOI:** 10.1002/ctm2.70377

**Published:** 2025-06-18

**Authors:** Lin Lu, Lei Xia, Xinhong Shi, Yingying Dai, Zipeng Wu, Yue Shi, Yingying Jiang, Yiling Liu, Yuxin Ma, Shuyi Hu, Ruofan Yu, Tianyi Liu, Caolu Liu, Jingwen Li, Guochun Cao, Delin Liu, Fei Yan, Jinghua Zhu, Xiaohua Wang, Lijun Zhao, Cheng Chen

**Affiliations:** ^1^ Department of Radiation Oncology The affiliated Cancer Hospital of Nanjing Medical University, Jiangsu Cancer Hospital, Jiangsu Institute of Cancer Research Nanjing China; ^2^ Department of Pathology The affiliated Cancer Hospital of Nanjing Medical University, Jiangsu Cancer Hospital, Jiangsu Institute of Cancer Research Nanjing China; ^3^ Department of Medical Oncology The Affiliated Cancer Hospital of Nanjing Medical University, Jiangsu Cancer Hospital, Jiangsu Institute of Cancer Research Nanjing China; ^4^ Department of Oncology Geriatric Hospital of Nanjing Medical University Nanjing China; ^5^ Department of Oncology Sichuan Second Hospital of Traditional Chinese Medicine Chengdu China; ^6^ Nanjing Medical University Nanjing China

1

Dear Editor,

We present the longest follow‐up to date (45.4 months) evaluating the survival outcomes of adding neoadjuvant immunochemotherapy (nICT) to chemotherapy (nCT) in resectable esophageal squamous cell carcinoma (ESCC). Our study showed that nICT improves event‐free survival (EFS) but does not significantly affect overall survival (OS), with residual viable tumour percentage (RVT%) potentially serving as a prognostic biomarker.

ESCC is the predominant form of esophageal cancer, with nCT or chemoradiotherapy being standard for resectable, locally advanced cases.^1^, ^2^, ^3^, ^4^ However, postoperative recurrence and metastasis remain major causes of failure.^5^ Recent Phase III trials reported that combining immune checkpoint inhibitors with chemotherapy may improve pathological complete response (pCR) rates,^6^, ^7^ though the long‐term survival benefit of nICT is unclear. Thus, we undertook this study.

We evaluated a total of 5472 patients diagnosed with ESCC and treated at Jiangsu Cancer Hospital between 2019 and 2021, with 125 patients in the nICT group and 132 in the nCT group. A 1:1 propensity score matching (PSM) was performed to minimize the impact of confounding factors. After matching, 206 patients were included, with 103 patients in each subgroup (see Supplementary Methods for details; Figure S1). Post‐PSM analysis indicated no significant differences in covariates between the groups (*p* > .05 for all), confirming a good balance in propensity scores (Table S1). Surgical approaches and short‐term postoperative outcomes were comparable between groups (Table S2).

Following PSM, the nICT and nCT groups exhibited identical R0 resection rates (99.0% each). The nICT group had a higher major pathological response (MPR) rate (49.5% vs. 31.1%, *p = *.007) and a lower median RVT% (11.0% [interquartile range {IQR} = 0.0–56.7] vs. 37.6% [IQR = 4.3–86.5], *p = *.003) (Figure 1A,B). The nICT group also demonstrated greater pathological regression in the primary tumour (*p = *.028), although the pCR rate did not differ significantly between the groups (23.3% vs. 13.6%, *p = *.072). Similarly, no significant differences were found in the rates of primary tumour downstaging (64.1% vs. 54.4%, *p = *.120) or lymph node downstaging (73.8% vs. 66.0%, *p = *.224) (Table S3). For cT1‐2 patients, RVT% was similar (*p = *.760), but in cT3‐4a patients, nICT showed significantly lower RVT% (*p = *.001) (Figure 1C).

**FIGURE 1:**
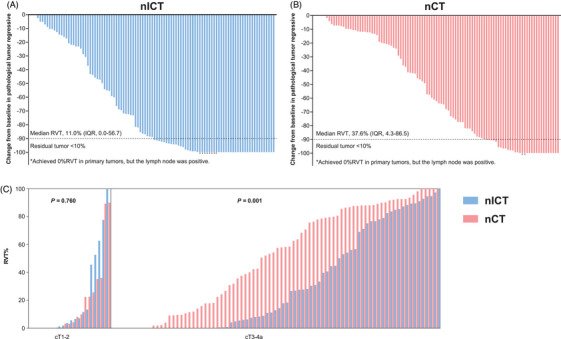
Depth of pathological response in the primary tumour. (A) The nICT group; (B) The nCT group; (C) The RVT% differences in the two groups by baseline stage of the disease. IQR, interquartile range; nCT, neoadjuvant chemotherapy; nICT, neoadjuvant immunochemotherapy RVT, residual viable tumour.

With a median follow‐up of 45.4 months (IQR = 24.9‐49.8), the median EFS was not reached in the nICT group, compared to 24.3 months in the nCT group (hazard ratio [HR] = 0.644, 95% confidence interval [95%CI] = 0.433–0.956, *p = *.029). The 1‐, 2‐, and 3‐year EFS rates were 78.6%, 63.1%, and 59.2% in the nICT group, compared to 72.8%, 51.5%, and 44.6% in the nCT group (Figure 2A). Although the median OS was unreached in both groups (HR = 0.758, 95%CI = 0.467–1.235; *p = *.270), the nICT group showed numerically higher 3‐year OS rates (73.7% vs. 66.9%), though this difference did not reach statistical significance (Figure 2B).

**FIGURE 2:**
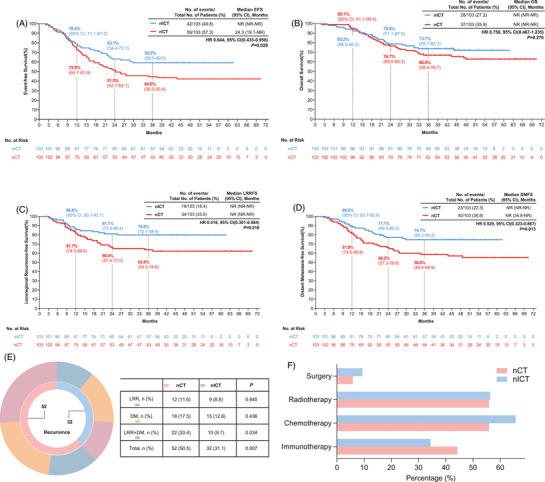
Survival outcomes and failure patterns after PSM. (A) Event‐free survival; (B) Overall survival; (C) Locoregional recurrence‐free survival; (D) Distant metastasis‐free survival; (E) Comparison of failure patterns. (F) Patterns of post‐recurrence salvage therapy (all *p* > .05). Cl, confidence interval; DMFS, distant metastasis‐free survival; EFS, event‐free survival; HR, hazards ratio; LRRFS, locoregional recurrence‐free survival; nCT, neoadjuvant chemotherapy; nlCT, neoadjuvant immunochemotherapy; NR, not reached; OS, overall survival; PSM, propensity score matching.

To explore this discrepancy, we analyzed recurrence patterns and post‐recurrence outcomes. The nICT regimen significantly reduced recurrence risk (31.1% vs. 50.5%, *p = *.007) (Figure 2E). Among patients who relapsed, 93.7% in the nICT group and 94.1% in the nCT group experienced recurrence within 2 years, and no significant differences were observed across the ≤12, 12–24, and 24–36 month intervals (Table S4). Post‐recurrence treatment patterns were also comparable, with most patients receiving salvage therapy—including chemotherapy, radiotherapy, immunotherapy, or their combinations—without significant intergroup differences (Figure 2F). Consistently, post‐recurrence survival was comparable between groups (*p = *.959; Figure S2), suggesting that similar salvage treatment strategies may have contributed to the lack of OS benefit despite improved early outcomes with nICT.

Notably, the nICT group achieved significant improvements in both locoregional control and systemic disease prevention. Although median locoregional recurrence‐free survival (LRFFS) and distant metastasis‐free survival (DMFS) were not reached in either arm, the nICT group showed significantly improved LRFFS (HR = 0.516, 95%CI = 0.301–0.884, *p = *.018) and DMFS (HR = 0.529, 95%CI = 0.323–0.867, *p = *.013) compared to the nCT group (Figure 2C,D).

To identify independent predictors of these clinical benefits, we performed comprehensive statistical analyses. Univariate analysis indicated that neoadjuvant therapy was a significant predictor of pCR, MPR, RVT%, EFS, and OS outcomes (Tables S5–9). All significant factors identified in the univariate analysis were subsequently included in multivariate regression models. Multivariate analysis confirmed nICT independently predicted improved pathological outcomes: higher pCR (OR = 2.361, 95%CI = 1.097–5.318), MPR (OR = 2.669, 95%CI = 1.434–5.104), and lower RVT% (OR = 2.132, 95%CI = 1.171–3.949). It prolonged EFS (HR = 0.610, 95%CI = 0.409–0.910, *p = *.015) but not OS (HR = 0.763, 95%CI = 0.464–1.255, *p = *.287) (Table 1). Subgroup analysis revealed a contrasting effect of adjuvant therapy on OS. Among patients who received adjuvant chemotherapy, no significant difference was observed in OS between the two groups. However, among those who did not receive adjuvant chemotherapy, nICT was associated with improved OS compared to nCT (HR = 0.432, 95%CI = 0.220–0.845, *p*‐interaction = 0.009). A similar trend was observed for adjuvant radiotherapy (HR = 0.510, 95%CI = 0.279–0.932, *p*‐interaction = 0.013) (Figure S3). The OS benefit of nICT in non‐adjuvant patients suggests immunotherapy may obviate the need for postoperative chemotherapy—a paradigm requiring validation in de‐escalation trials.

**TABLE 1:** Association between neoadjuvant therapy and efficacies in univariate and multivariate analyses after propensity score matching.

	No. of events/no. of patients at risk (%)					
Efficacy	nCT	nICT	Univariate Analysis 95%CI	*p*	Multivariate analysis^a^ 95%CI	*p*
pCR	14/103 (13.6)	24/103 (23.3)	1.931 (0.935–3.989)	.072	2.361 (1.097–5.318)	.032
MPR	32/103 (31.1)	51/103 (49.5)	2.176 (1.232–3.843)	.007	2.669 (1.434–5.104)	.002
RVT<25.02%^b^	44/103 (42.7)	59/103 (57.3)	1.798 (1.035–3.123)	.037	2.132 (1.171–3.949)	.014
EFS	58/103 (56.3)	42/103 (40.8)	0.644 (0.433–0.956)	.029	0.610 (0.409–0.910)	.015
OS	34/103 (33.0)	29/103 (28.2)	0.758 (0.467–1.235)	.270	0.763 (0.464–1.255)	.287

Abbreviations: CI: confidence interval; EFS, event‐free survival; MPR, major pathological response; nICT, neoadjuvant immunochemotherapy; OS: overall survival; pCR, pathological complete response; RVT, residual viable tumour.

^a^ Adjusted for sex, age, smoking history, alcohol use history, tumour location, cT, cN, and treatment cycles.

^b^ Median RVT.nCT: neoadjuvant chemotherapy.

To further characterize prognostic determinants, we examined the association between RVT% and survival outcomes across treatment groups. With mortality data updated through July 2024 (65 deaths; mortality rate 31.6%), RVT% exhibited a non‐linear relationship with survival (*p* for non‐linearity = 0.034) (Figure 3A). A critical threshold at 25.02% optimally stratified risk: below this cutoff, each standard deviation (SD) increase in RVT% significantly elevated mortality risk (HR = 1.100 per SD, 95%CI = 1.030–1.170, *p = *.003), whereas above 25.02%, RVT% increments showed no significant association (HR = 1.010 per SD, 95%CI = 0.940–1.020, *p = *.203) (Table S10). Patients with RVT% ≥25.02% had markedly worse OS versus those below the threshold (log‐rank *p *< 0.001) (Figure 3B). This prognostic dichotomy persisted in both the nICT (HR = 0.400, 95%CI = 0.187–0.858, *p = *.014) and nCT groups (HR = 0.415, 95%CI = 0.218–0.792, *p = *.014) (Figure 3C,D).

**FIGURE 3:**
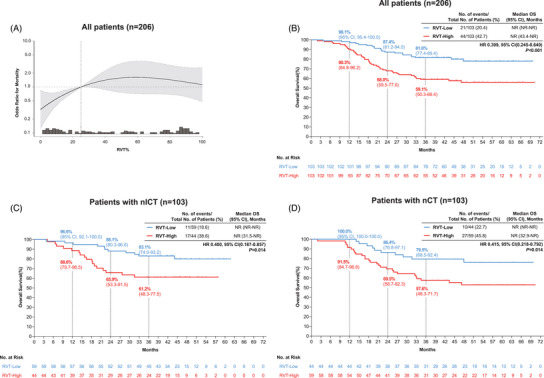
Association RVT% with OS stratified by treatment group after PSM. (A) Association of RVT% with the risk of mortality (*p* for non‐linearity:0.034). (B) OS by RVT% in all patients; (C) OS by RVT% in patients receiving nICT; (D) OS by RVT% in patients receiving nCT. CI, confidence interval; HR, hazards ratio; nICT, neoadjuvant immunochemotherapy; nCT, neoadjuvant chemotherapy; NR, not reached; PSM, propensity score matching; RVT, Residual viable tumour (RVT‐Low: RVT% < 25.02%, RVT‐High: RVT ≥ 25.02).

In conclusion, our study indicates that neoadjuvant immunochemotherapy improves pathological response and EFS in resectable ESCC but shows no significant OS benefit over chemotherapy. Further research should validate RVT% as a prognostic marker and explore biomarkers to optimize patient selection, aiming for more personalized and effective treatment strategies.

## ETHICS STATEMENT

Ethical approval for this study (Ethical Committee N° KY‐2024‐138) was provided by the Ethical Committee of Jiangsu Cancer Hospital. Informed consent was waived due to the retrospective nature of the study.

## Supporting information



Supporting Information
