# Supplementary material for: Long‐term survival and association of neoadjuvant chemotherapy with or without immunotherapy in resectable esophageal squamous cell carcinoma
Source: Clin Transl Med. 2025 Jun 18;15(6):e70377. doi: 10.1002/ctm2.70377 (PMC12177103; doi:10.1002/ctm2.70377)
Supplement: Supplementary file 1 — Supporting Information [file CTM2-15-e70377-s001.docx]

**Supplementary Materials to: Long-Term Survival and Association of Neoadjuvant Chemotherapy with or without Immunotherapy in Resectable Eesophageal Squamous Cell Carcinoma**

This Appendix provides further methodological details and additional Tables and Figures for “Long-Term Survival and Association of Neoadjuvant Chemotherapy with or without Immunotherapy in Resectable Esophageal Squamous Cell Carcinoma”

Contents

| Supplementary Methods |  |
| --- | --- |
| Patient sample and data collection | page 2 |
| Treatment Regimens | page 2 |
| Radiological assessment and Follow-up | page 3 |
| Pathological Assessment | page 3 |
| Statistical analysis | page 3 |
| Supplementary Figures and Tables |  |
| Figure S1 | page 5 |
| Figure S2 | page 6 |
| Figure S3 | page 7 |
| Table S1 | page 8 |
| Table S2 | page 10 |
| Table S3 | page 11 |
| Table S4 | page 13 |
| Table S5 | page 14 |
| Table S6 | page 15 |
| Table S7 | page 16 |
| Table S8 | page 17 |
| Table S9 | page 18 |
| Table S10 | page 19 |
| References | page 20 |

Supplementary Methods

**Patient sample and data collection**

This study analysed patients with resectable ESCC treated at Jiangsu Cancer Hospital between January 2019 and December 2021, with the last follow-up conducted on 31 July 2024. The inclusion criteria were as follows: (I) Pathologically confirmed ESCC; (II) Clinical staging as cT1N+M0 or cT2-4aNanyM0 thoracic esophageal cancer, according to the 8th edition of the American Joint Committee on Cancer (AJCC) TNM staging system; (III) Receipt of nCT or nICT followed by curative esophagectomy. Patients were excluded if they: (I) Failed to meet the specified staging criteria or treatment protocols; (II) Presented with histological subtypes other than squamous cell carcinoma; (III) Had a history of autoimmune diseases, severe cardiovascular conditions, or other malignancies; (III) Lacked essential information, such as preoperative imaging, drug regimens, or other clinical records. Ethical approval for this study (Ethical Committee N° KY-2024-138) was provided by the Ethical Committee of Jiangsu Cancer Hospital. Informed consent was waived due to the retrospective nature of the study. All patient data were anonymised to ensure confidentiality.

**Treatment Regimens**

In the nICT group, common regimens included sintilimab (200 mg), paclitaxel (135 mg/m²), and carboplatin (area under the curve [AUC]=5) administered on day 1 of each cycle; or toripalimab (240 mg), paclitaxel (135 mg/m²), and carboplatin (AUC=5) administered on day 1. In the nCT group, the most common regimen was TP: paclitaxel (135 mg/m²) and carboplatin (AUC=5) administered on day 1. Neoadjuvant therapy was given every three weeks for a total of 2–4 cycles.

Following neoadjuvant therapy, patients generally observed a rest period of 4 to 8 weeks prior to undergoing esophagectomy. All patients underwent one of several esophagectomy types, which included standard lymphadenectomy via open or minimally invasive transthoracic or transhiatal approaches. The three most frequently employed procedures were the Sweet procedure, the Ivor-Lewis procedure, and the McKeown procedure. Surgical parameters and short-term outcomes, including blood loss, operative time, length of postoperative hospital stay, and 30- and 90-day postoperative mortality rates, were compared between the two groups. In the nICT group, adjuvant immunotherapy was administered for one-year post-surgery.

**Radiological assessment and Follow-up**

Following neoadjuvant therapy, enhanced CT scans, endoscopic ultrasound, and esophagography were performed. Radiographic responses were independently evaluated by two senior radiologists using the Response Evaluation Criteria in Solid Tumors (RECIST 1.1) ^[1]^. Clinical staging was conducted based on the 8th edition of the AJCC TNM staging system for esophageal cancer. Recurrence at the esophageal or anastomotic site was evaluated using endoscopic ultrasound. Metastatic lymph nodes, as well as suspected metastases in the liver, brain, or bone, were further investigated with liver magnetic resonance imaging (MRI), brain MRI, and bone scintigraphy. Event-free survival (EFS) was defined as the duration from the start of treatment to disease progression, postoperative recurrence, or death from any cause. Overall survival (OS) was calculated from the start of treatment to the occurrence of death from any cause. the day of diagnosis to the day of to the occurrence of death from any cause. Locoregional recurrence (LRR) was characterized as recurrence within the esophagus, at the anastomotic site, or in regional lymph nodes. Distant metastasis (DM) was defined as recurrence involving nonregional lymph nodes (e.g., supraclavicular or para-aortic nodes), peritoneal carcinomatosis, or metastases to distant organs. Post-recurrence survival (PRS) was defined as the time from the date of first confirmed recurrence to death or last follow-up. The follow-up period was measured from the date of surgery to either the date of death or the most recent follow-up.

**Pathological Assessment**

In this study, all pathological samples were reviewed, and tumor regression grade was evaluated in accordance with the Becker criteria^[2]^. The percentage of residual viable tumor (RVT%) was calculated by comparing the residual tumor area to the total area of residual tumor, stroma, and necrosis^[3]^. A pathological complete response (pCR) was characterized by the absence of residual tumor cells in both the primary lesion and lymph nodes, whereas a major pathological response (MPR) was defined as a residual tumor proportion of less than 10%. All pathological data were independently reviewed by two pathologists.

**Statistical analysis**

Categorical variables between the nICT and nCT groups were compared with Chi-square tests. Parametric continuous data were analysed using Student's t-tests, and nonparametric continuous data with the Mann-Whitney U test. Kaplan-Meier analyses with log-rank tests assessed differences in EFS, OS, locoregional recurrence-free survival (LRRFS), and distant metastasis-free survival (DMFS). A Cox proportional hazards model adjusted for confounders and identify independent factors impacting EFS and OS. Multiple logistic regression was performed to determine independent factors influencing treatment outcomes. Threshold levels (turning points) for continuous variables were determined using likelihood-ratio tests and bootstrap resampling methods. Nearest-neighbour 1:1 propensity score matching (PSM) with a caliper of 0.02 was performed to minimise confounding bias between the nICT and nCT groups. Propensity scores were calculated using a multivariable logistic regression model that included age, sex, smoking history, alcohol use history, tumor localisation, clinical T stage, clinical N stage, and the number of neoadjuvant treatment cycles. Statistical analyses were performed using SPSS version 26.0 (SPSS Inc., Chicago, IL), while PSM was conducted in R version 4.3.1. Graphs were generated using GraphPad Prism version 9.5.1. Statistical significance was determined by a *P*-value of 0.05.

Supplementary Figures and Tables





**Figure S1. Flow diagram of the patient selectlon process.**

nCRT: neoadjuvant chemoradiotherapy; nICT: neoadjuvant immunochemotherapy; nCT: neoadjuvant chemotherapy.


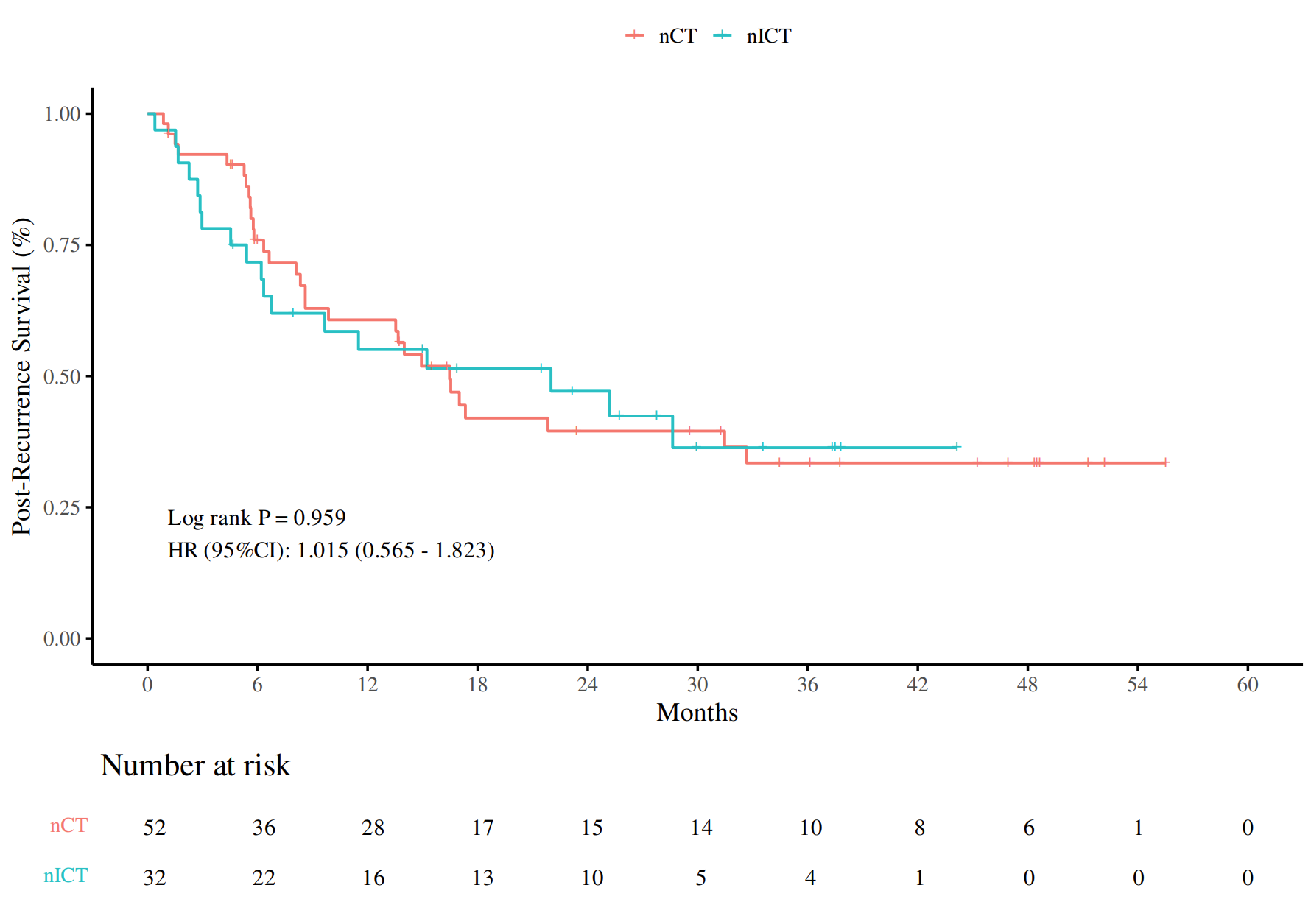


**Figure S2. Kaplan-Meier Curves for Post-Recurrence Survival in nCT and nICT Groups.**

nlCT: neoadjuvant immunochemotherapy; nCT: neoadjuvant chemotherapy; HR: hazard ratio; Cl: confidence interval.





**Figure S3. Forest plot of subgroup analysis for overall survival after propensity score matching.**

nlCT: neoadjuvant immunochemotherapy; nCT: neoadjuvant chemotherapy; HR: hazard ratio; Cl: confidence interval.

| Table S1. Clinical characteristics of patients before and after propensity score matching. | | | | | | | | | |
| --- | --- | --- | --- | --- | --- | --- | --- | --- | --- |
| **Characteristic** |  | **Unmatched Patients** | | | |  | **PSM Patients** | | |
|  | nICT | | nCT | *P* | nICT | | | nCT | *P* |
|  | n=125 | | n=132 |  | n=103 | | | n=103 |  |
|  | *n, (%)* | | |  | *n, (%)* | | | |  |
| SEX |  | |  |  |  | | |  |  |
| MALE | 98 (78.4) | | 110 (83.3) | 0.397 | 84 (81.6) | | | 83 (80.6) | 1.000 |
| FEMALE | 27 (21.6) | | 22 (16.7) |  | 19 (18.4) | | | 20 (19.4) |  |
| AGE |  | |  |  |  | | |  |  |
| ≤60 | 36 (28.8) | | 37 (28.0) | 1.000 | 29 (28.2) | | | 27 (26.2) | 0.876 |
| ＞60 | 89 (71.2) | | 95 (72.0) |  | 74 (71.8) | | | 76 (73.8) |  |
| SMOKING |  | |  |  |  | | |  |  |
| YES | 24 (19.2) | | 27 (20.5) | 0.924 | 20 (19.4) | | | 23 (22.3) | 0.732 |
| NO | 101 (80.8) | | 105 (79.5) |  | 83 (80.6) | | | 80 (77.7) |  |
| ALCOHOL |  | |  |  |  | | |  |  |
| YES | 24 (19.2) | | 24 (18.2) | 0.961 | 19 (18.4) | | | 19 (18.4) | 1.000 |
| NO | 101 (80.8) | | 108 (81.8) |  | 84 (81.6) | | | 84 (81.6) |  |
| LOCATION |  | |  |  |  | | |  |  |
| UPPER | 11 (8.8) | | 14 (10.6) | 0.739 | 6 (5.8) | | | 10 (9.7) | 0.580 |
| MIDDLE | 72 (57.6) | | 79 (59.8) |  | 61 (59.2) | | | 58 (56.3) |  |
| DISTAL | 42 (33.6) | | 39 (29.5) |  | 36 (35.0) | | | 35 (34.0) |  |
| cT |  | |  |  |  | | |  |  |
| T1-2 | 30 (24.0) | | 23 (17.4) | 0.251 | 20 (19.4) | | | 21 (20.4) | 1.000 |
| T3-4a | 95 (76.0) | | 109 (82.6) |  | 83 (80.6) | | | 82 (79.6) |  |
| cN |  | |  |  |  | | |  |  |
| N0 | 9 (7.2) | | 12 (9.1) | 0.499 | 8 (7.8) | | | 11 (10.7) | 0.769 |
| N1 | 54 (43.2) | | 64 (48.5) |  | 44 (42.7) | | | 43 (41.7) |  |
| N2-3 | 62 (49.6) | | 56 (42.4) |  | 51 (49.5) | | | 49 (47.6) |  |
| cTNM |  | |  |  |  | | |  |  |
| I-II | 24 (19.2) | | 26 (19.7) | 1.000 | 20 (19.4) | | | 23 (22.3) | 0.732 |
| III-IVa | 101 (80.8) | | 106 (80.3) |  | 83 (80.6) | | | 80 (77.7) |  |
| CYCLE |  | |  |  |  | | |  |  |
| 2 | 102 (81.6) | | 120 (90.9) | 0.030 | 93 (90.3) | | | 92 (89.3) | 0.818 |
| 3-4 | 23 (18.4) | | 12 (9.1) |  | 10 (9.7) | | | 11 (10.7) |  |
| PSM: propensity score matching; nICT: neoadjuvant immunochemotherapy; nCT: neoadjuvant chemotherapy. | | | | | | | | | |

| Table S2. Comparative analysis of surgical procedures and short-term outcomes before and after propensity score matching. | | | | | | | | |
| --- | --- | --- | --- | --- | --- | --- | --- | --- |
| **Characteristic** | **Unmatched Patients** | |  | **PSM Patients** | | |  |  |
|  | nICT | nCT | *P* | nICT | nCT | *P* | |  |
|  | n=125 | n=132 |  | n=103 | n=103 |  | |  |
| Types of surgical procedures, n (%) |  |  |  |  |  |  | |  |
| Sweet | 10(8.0) | 6(4.5) | 0.246 | 6(5.8) | 5(4.8) | 0.699 | |  |
| Ivor-lewis | 67(53.6) | 83(62.9) |  | 54(52.4) | 60(58.3) |  | |  |
| McKeown | 48(38.4) | 43(32.6) |  | 43(41.8) | 38(36.9) |  | |  |
| Surgical approach, n (%) |  |  |  |  |  |  | |  |
| MIE | 58(46.4) | 58(43.9) | 0.787 | 49(47.6) | 44(42.7) | 0.575 | |  |
| OE | 67(53.6) | 74(56.1) |  | 54(52.4) | 59(57.3) |  | |  |
| Operative time(minute)^a^ | 260.7±62.4 | 289.1±78.1 | 0.002 | 259.7±59.6 | 287.5±76.7 | 0.004 | |  |
| Bleeding volume(ml)^a^ | 186.0±216.5 | 234.9±539.5 | 0.347 | 182.0±229.5 | 250.2±608.4 | 0.289 | |  |
| Blood transfusion during hospitalization, n (%) |  |  |  |  |  |  | |  |
| YES | 18(14.4) | 21(15.9) | 0.736 | 13(12.6) | 18(17.5) | 0.330 | |  |
| NO | 107(85.6) | 111(84.1) |  | 90(87.4) | 85(82.5) |  | |  |
| ICU, n (%) |  |  |  |  |  |  | |  |
| YES | 83(66.4) | 112(84.8) | 0.001 | 68(66.0) | 84(81.6) | 0.011 | |  |
| NO | 42(33.6) | 20(15.2) |  | 35(34.0) | 19(18.4) |  | |  |
| Postoperative hospital stay (days)^a^ | 15.1±11.6 | 16.4±13.9 | 0.441 | 15.0±10.6 | 15.6±8.7 | 0.667 | |  |
| 30-day mortality, n (%) | 2(1.6) | 1(0.8) | 0.530 | 1(1.0) | 1(1.0) | 1.000 | |  |
| 90-day mortality, n (%) | 4(3.2) | 1(0.8) | 0.157 | 3(2.9) | 1(1.0) | 0.313 | |  |
| ^a^: Data are presented as mean ± standard deviation.  PSM: propensity score matching; nICT: neoadjuvant immunochemotherapy; nCT: neoadjuvant chemotherapy; MIE: minimally invasive esophagectomy; OE: open esophagectomy; ICU: intensive care unit. | | | | | | | | |

| Table S3. Pathological outcomes and downstaging after propensity score matching. | | | | |
| --- | --- | --- | --- | --- |
| **Pathologic characteristics** | | nICT | nCT | *P* |
|  |  | n=103 | n=103 |  |
| R0 resection, n (%) |  |  |  |  |
| R0 |  | 102(99.0) | 102(99.0) | 1.000 |
| R1 |  | 1(1.0) | 1(1.0) |  |
| pCR , n (%) |  |  |  |  |
| Yes |  | 24(23.3) | 14(13.6) | 0.072 |
| No |  | 79(76.7) | 89(86.4) |  |
| MPR, n (%) |  |  |  |  |
| Yes |  | 51(49.5) | 32(31.1) | 0.007 |
| No |  | 52(50.5) | 71(68.9) |  |
| RVT^a^ |  | 11.0(0.0-56.7) | 37.6(4.3-86.5) | 0.003 |
| TRG, n (%) |  |  |  |  |
| TRG1a |  | 32(31.1) | 16(15.5) | 0.028 |
| TRG1b |  | 19(18.4) | 16(15.5) |  |
| TRG2 |  | 21(20.4) | 24(23.3) |  |
| TRG3 |  | 31(30.1) | 47(45.7) |  |
| T downstaging, n (%) |  |  |  |  |
| Yes |  | 66(64.1) | 56(54.3) | 0.120 |
| No |  | 37(35.9) | 47(45.7) |  |
| N downstaging, n (%) |  |  |  |  |
| Yes |  | 76(73.8) | 68(66.0) | 0.224 |
| No |  | 27(26.2) | 35(34.0) |  |
| ypT, n (%) |  |  |  |  |
| ypT0 |  | 23(22.3) | 9(8.7) | 0.008 |
| ypTis |  | 9(8.7) | 7(6.8) |  |
| ypT1a |  | 8(7.8) | 1(1.0) |  |
| ypT1b |  | 9(8.7) | 10(9.7) |  |
| ypT2 |  | 19(18.4) | 27(26.2) |  |
| ypT3 |  | 35(34.0) | 49(47.6) |  |
| ypN, n (%) |  |  |  |  |
| ypN0 |  | 62(60.2) | 54(52.4) | 0.727 |
| ypN1 |  | 27(26.2) | 33(32.1) |  |
| ypN2 |  | 10(9.7) | 11(10.7) |  |
| ypN3 |  | 4(3.9) | 5(4.8) |  |
| ypTNM, n (%) |  |  |  |  |
| I |  | 43(41.8) | 34(33.0) | 0.375 |
| II |  | 19(18.4) | 20(19.4) |  |
| IIIA |  | 19(18.4) | 15(14.6) |  |
| IIIB |  | 18(17.5) | 29(28.2) |  |
| IVA |  | 4(3.9) | 5(4.8) |  |
| ^a^: Data are presented as median (interquartile range).  nICT: neoadjuvant immunochemotherapy; nCT: neoadjuvant chemotherapy; pCR: pathological complete response; MPR: major pathological response; RVT: residual viable tumor; TRG: tumor regression grade. | | | | |

| Table S5. Univariate and multivariate logistic regression analyses for pathological complete response after propensity score matching. | | | | | | |
| --- | --- | --- | --- | --- | --- | --- |
| Characteristics |  | Univariate | *P* |  | Multivariate | *P* |
|  |  | OR(95%CI) |  |  | OR(95%CI) |  |
| GROUP |  |  |  |  |  |  |
| nCT |  |  | Reference |  |  | Reference |
| nICT |  | 1.931(0.935-3.989) | 0.072 |  | 2.361(1.097-5.318) | 0.032 |
| SEX |  |  |  |  |  |  |
| MALE |  |  | Reference |  |  | Reference |
| FEMALE |  | 0.597(0.217-1.645) | 0.319 |  | 0.557(0.159-1.645) | 0.318 |
| AGE |  |  |  |  |  |  |
| ≤60 |  |  | Reference |  |  | Reference |
| >60 |  | 1.252(0.551-2.842) | 0.592 |  | 1.219(0.516-3.098) | 0.661 |
| SMOKING |  |  |  |  |  |  |
| YES |  |  | Reference |  |  | Reference |
| NO |  | 0.578(0.260-1.285) | 0.179 |  | 0.207(0.048-0.857) | 0.030 |
| ALCOHOL |  |  |  |  |  |  |
| YES |  |  | Reference |  |  | Reference |
| NO |  | 1.002(0.404-2.485) | 0.996 |  | 4.264(0.916-21.889) | 0.071 |
| LOCATION |  |  |  |  |  |  |
| UPPER |  |  | Reference |  |  | Reference |
| MIDDLE |  | 0.499(0.157-1.582) | 0.238 |  | 0.464(0.135-1.760) | 0.233 |
| DISTAL |  | 0.403(0.117-1.390) | 0.150 |  | 0.350(0.092-1.436) | 0.129 |
| cT |  |  |  |  |  |  |
| T1-2 |  |  | Reference |  |  | Reference |
| T3-4a |  | 0.452(0.205-0.998) | 0.050 |  | 0.463(0.191-1.151) | 0.091 |
| cN |  |  |  |  |  |  |
| N0 |  |  | Reference |  |  | Reference |
| N1 |  | 1.805(0.480-6.788) | 0.382 |  | 1.994(0.516-10.299) | 0.355 |
| N2-3 |  | 0.797(0.204-3.117) | 0.744 |  | 0.879(0.223-4.518) | 0.863 |
| CYCLE |  |  |  |  |  |  |
| 2 |  |  | Reference |  |  | Reference |
| 3-4 |  | 1.913(0.689-5.307) | 0.213 |  | 2.626(0.793-8.083) | 0.098 |
| nCT: neoadjuvant chemotherapy; nICT: neoadjuvant immunochemotherapy; OR: odds ratio; CI: confidence interval. | | | | | | |

| Table S6. Univariate and multivariate logistic regression analyses for major pathological response after propensity score matching. | | | | | | |
| --- | --- | --- | --- | --- | --- | --- |
| Characteristics |  | Univariate | *P* |  | Multivariate | *P* |
|  |  | OR(95%CI) |  |  | OR(95%CI) |  |
| GROUP |  |  |  |  |  |  |
| nCT |  |  | Reference |  |  | Reference |
| nICT |  | 2.176(1.232-3.843) | 0.007 |  | 2.669(1.434-5.104) | 0.002 |
| SEX |  |  |  |  |  |  |
| MALE |  |  | Reference |  |  | Reference |
| FEMALE |  | 0.795(0.386-1.639) | 0.535 |  | 0.686(0.278-1.627) | 0.400 |
| AGE |  |  |  |  |  |  |
| ≤60 |  |  | Reference |  |  | Reference |
| >60 |  | 1.174(0.625-2.207) | 0.618 |  | 1.099(0.550-2.224) | 0.790 |
| SMOKING |  |  |  |  |  |  |
| YES |  |  | Reference |  |  | Reference |
| NO |  | 0.817(0.414-1.610) | 0.559 |  | 0.329(0.089-1.095) | 0.077 |
| ALCOHOL |  |  |  |  |  |  |
| YES |  |  | Reference |  |  | Reference |
| NO |  | 1.195(0.577-2.473) | 0.631 |  | 4.014(1.120-16.284) | 0.039 |
| LOCATION |  |  |  |  |  |  |
| UPPER |  |  | Reference |  |  | Reference |
| MIDDLE |  | 0.378(0.129-1.110) | 0.077 |  | 0.402(0.117-1.294) | 0.131 |
| DISTAL |  | 0.368(0.120-1.128) | 0.080 |  | 0.396(0.110-1.350) | 0.142 |
| cT |  |  |  |  |  |  |
| T1-2 |  |  | Reference |  |  | Reference |
| T3-4a |  | 0.266(0.129-0.548) | <0.001 |  | 0.205(0.088-0.453) | <0.001 |
| cN |  |  |  |  |  |  |
| N0 |  |  | Reference |  |  | Reference |
| N1 |  | 4.136(1.123-15.236) | 0.033 |  | 4.030(0.977-20.167) | 0.055 |
| N2-3 |  | 3.862(1.057-14.108) | 0.041 |  | 4.849(1.329-23.996) | 0.028 |
| CYCLE |  |  |  |  |  |  |
| 2 |  |  | Reference |  |  | Reference |
| 3-4 |  | 0.903(0.357-2.284) | 0.829 |  | 1.409(0.479-4.027)) | 0.523 |
| nCT: neoadjuvant chemotherapy; nICT: neoadjuvant immunochemotherapy; OR: odds ratio; CI: confidence interval. | | | | | | |

| Table S7. Univariate and multivariate logistic regression analyses for residual viable tumor after propensity score matching. | | | | | | |
| --- | --- | --- | --- | --- | --- | --- |
| Characteristics |  | Univariate | *P* |  | Multivariate | *P* |
|  |  | OR(95%CI) |  |  | OR(95%CI) |  |
| GROUP |  |  |  |  |  |  |
| nCT |  |  | Reference |  |  | Reference |
| nICT |  | 1.798(1.035-3.123) | 0.037 |  | 2.132(1.171-3.949) | 0.014 |
| SEX |  |  |  |  |  |  |
| MALE |  |  | Reference |  |  | Reference |
| FEMALE |  | 0.728(0.361-1.468) | 0.375 |  | 0.671(0.286-1.539) | 0.350 |
| AGE |  |  |  |  |  |  |
| ≤60 |  |  | Reference |  |  | Reference |
| >60 |  | 1.217(0.658-2.251) | 0.531 |  | 1.171(0.597-2.309) | 0.647 |
| SMOKING |  |  |  |  |  |  |
| YES |  |  | Reference |  |  | Reference |
| NO |  | 0.745(0.379-1.463) | 0.392 |  | 0.296(0.075-0.976) | 0.058 |
| ALCOHOL |  |  |  |  |  |  |
| YES |  |  | Reference |  |  | Reference |
| NO |  | 1.138(0.562-2.303) | 0.719 |  | 4.194(1.197-17.569) | 0.033 |
| LOCATION |  |  |  |  |  |  |
| UPPER |  |  | Reference |  |  | Reference |
| MIDDLE |  | 0.306(0.093-1.005) | 0.051 |  | 0.315(0.079-1.054) | 0.075 |
| DISTAL |  | 0.306(0.090-1.041) | 0.058 |  | 0.335(0.081-1.176) | 0.102 |
| cT |  |  |  |  |  |  |
| T1-2 |  |  | Reference |  |  | Reference |
| T3-4a |  | 0.250(0.115-0.543) | <0.001 |  | 0.205(0.084-0.462) | <0.001 |
| cN |  |  |  |  |  |  |
| N0 |  |  | Reference |  |  | Reference |
| N1 |  | 2.217(0.772-6.366) | 0.139 |  | 1.804(0.578-6.168) | 0.323 |
| N2-3 |  | 2.443(0.860-6.940) | 0.094 |  | 2.654(0.873-8.959) | 0.096 |
| CYCLE |  |  |  |  |  |  |
| 2 |  |  | Reference |  |  | Reference |
| 3-4 |  | 0.726(0.292-1.806) | 0.491 |  | 0.977(0.348-2.693) | 0.965 |
| nCT: neoadjuvant chemotherapy; nICT: neoadjuvant immunochemotherapy; OR: odds ratio; CI: confidence interval. | | | | | | |

| Table S8. Univariate and multivariate COX regression analyses for event-free survival after propensity score matching. | | | | | | |
| --- | --- | --- | --- | --- | --- | --- |
| Characteristics |  | Univariate | *P* |  | Multivariate | *P* |
|  |  | HR(95%CI) |  |  | HR(95%CI) |  |
| GROUP |  |  |  |  |  |  |
| nCT |  |  | Reference |  |  | Reference |
| nICT |  | 0.644(0.433-0.956) | 0.029 |  | 0.610(0.409-0.910) | 0.015 |
| SEX |  |  |  |  |  |  |
| MALE |  |  | Reference |  |  | Reference |
| FEMALE |  | 0.729(0.427-1.240) | 0.246 |  | 0.778(0.442-1.369) | 0.384 |
| AGE |  |  |  |  |  |  |
| ≤60 |  |  | Reference |  |  | Reference |
| >60 |  | 0.834(0.544-1.280) | 0.404 |  | 0.891(0.576-1.377) | 0.603 |
| SMOKING |  |  |  |  |  |  |
| YES |  |  | Reference |  |  | Reference |
| NO |  | 0.958(0.592-1.550) | 0.860 |  | 1.344(0.639-2.828) | 0.436 |
| ALCOHOL |  |  |  |  |  |  |
| YES |  |  | Reference |  |  | Reference |
| NO |  | 0.792(0.490-1.280) | 0.342 |  | 0.682(0.323-1.442) | 0.317 |
| LOCATION |  |  |  |  |  |  |
| UPPER |  |  | Reference |  |  | Reference |
| MIDDLE |  | 1.360(0.623-2.970) | 0.439 |  | 1.304(0.591-2.877) | 0.511 |
| DISTAL |  | 1.090(0.481-2.470) | 0.837 |  | 1.077(0.468-2.478) | 0.862 |
| cT |  |  |  |  |  |  |
| T1-2 |  |  | Reference |  |  | Reference |
| T3-4a |  | 1.700(0.985-2.950) | 0.057 |  | 1.543(0.873-2.726) | 0.135 |
| cN |  |  |  |  |  |  |
| N0 |  |  | Reference |  |  | Reference |
| N1 |  | 0.568(0.285-1.130) | 0.107 |  | 0.704(0.344-1.439) | 0.336 |
| N2-3 |  | 1.160(0.609-2.210) | 0.651 |  | 1.279(0.649-2.521) | 0.476 |
| CYCLE |  |  |  |  |  |  |
| 2 |  |  | Reference |  |  | Reference |
| 3-4 |  | 1.290(0.708-2.360) | 0.403 |  | 1.119(0.592-2.112) | 0.730 |
| nCT: neoadjuvant chemotherapy; nICT: neoadjuvant immunochemotherapy; HR: hazard ratio; CI: confidence interval. | | | | | | |

| Table S9. Univariate and multivariate COX regression analyses for overall survival after propensity score matching. | | | | | | |
| --- | --- | --- | --- | --- | --- | --- |
| Characteristics |  | Univariate | *P* |  | Multivariate | *P* |
|  |  | HR(95%CI) |  |  | HR(95%CI) |  |
| GROUP |  |  |  |  |  |  |
| nCT |  |  | Reference |  |  | Reference |
| nICT |  | 0.758(0.467-1.235) | 0.270 |  | 0.763(0.464-1.255) | 0.287 |
| SEX |  |  |  |  |  |  |
| MALE |  |  | Reference |  |  | Reference |
| FEMALE |  | 0.746(0.380-1.460) | 0.394 |  | 0.757(0.371-1.546) | 0.445 |
| AGE |  |  |  |  |  |  |
| ≤60 |  |  | Reference |  |  | Reference |
| >60 |  | 0.991(0.576-1.710) | 0.974 |  | 1.077(0.620-1.869) | 0.793 |
| SMOKING |  |  |  |  |  |  |
| YES |  |  | Reference |  |  | Reference |
| NO |  | 1.050(0.569-1.920) | 0.885 |  | 1.065(0.409-2.774) | 0.897 |
| ALCOHOL |  |  |  |  |  |  |
| YES |  |  | Reference |  |  | Reference |
| NO |  | 1.010(0.541-1.890) | 0.970 |  | 1.106(0.413-2.962) | 0.841 |
| LOCATION |  |  |  |  |  |  |
| UPPER |  |  | Reference |  |  | Reference |
| MIDDLE |  | 0.984(0.387-2.500) | 0.973 |  | 0.826(0.320-2.128) | 0.692 |
| DISTAL |  | 0.999(0.380-2.630) | 0.998 |  | 0.885(0.328-2.383) | 0.808 |
| cT |  |  |  |  |  |  |
| T1-2 |  |  | Reference |  |  | Reference |
| T3-4a |  | 1.730(0.857-3.500) | 0.126 |  | 1.536(0.745-3.165) | 0.245 |
| cN |  |  |  |  |  |  |
| N0 |  |  | Reference |  |  | Reference |
| N1 |  | 0.456(0.187-1.110) | 0.083 |  | 0.437(0.174-1.094) | 0.077 |
| N2-3 |  | 1.180(0.528-2.620) | 0.693 |  | 1.089(0.472-2.510) | 0.842 |
| CYCLE |  |  |  |  |  |  |
| 2 |  |  | Reference |  |  | Reference |
| 3-4 |  | 0.696(0.279-1.730) | 0.436 |  | 0.575(0.223-1.484) | 0.253 |
| nCT: neoadjuvant chemotherapy; nICT: neoadjuvant immunochemotherapy; HR: hazard ratio; CI: confidence interval. | | | | | | |

| Table S10. Association between residual viable tumor and mortality in esophageal squamous cell carcinoma. | | | | |
| --- | --- | --- | --- | --- |
| **RVT** | Unadjusted | | Adjusted^a^ | |
|  | HR (95%CI) | *P* for trend | HR (95%CI) | *P* for trend |
| Per SD increment | 1.010(1.010-1.020) | <0.001 | 1.010(1.010-1.020) | <0.001 |
| RVT groups |  |  |  |  |
| <25.02% | 1.070(1.020-1.130) | 0.011 | 1.100(1.030-1.170) | 0.003 |
| ≥25.02% | 1.000(0.990-1.020) | 0.539 | 1.010(0.940-1.020) | 0.203 |
| ^a^: Adjusted for sex, age, smoking history, alcohol use history, tumor location, cT, cN, and treatment cycles.  RVT: residual viable tumor; HR: hazard ratio; CI: confidence interval. | | | | |

References

1. Eisenhauer EA, Therasse P, Bogaerts J, et al. New response evaluation criteria in solid tumours: revised RECIST guideline (version 1.1). Eur J Cancer. 2009;45(2):228-247.
2. Becker K, Mueller JD, Schulmacher C, et al. Histomorphology and grading of regression in gastric carcinoma treated with neoadjuvant chemotherapy. Cancer. 2003;98(7):1521-1530.
3. Cottrell TR, Thompson ED, Forde PM, et al. Pathologic features of response to neoadjuvant anti-PD-1 in resected non-small-cell lung carcinoma: a proposal for quantitative immune-related pathologic response criteria (irPRC). Ann Oncol. 2018;29(8):1853-1860.
